# Data analysis of viral complementary DNA/RNA sequences for low-frequency intrahost–single nucleotide variants in COVID-19

**DOI:** 10.1073/pnas.2205269119

**Published:** 2022-08-01

**Authors:** Samarth Chandra

**Affiliations:** ^a^Spinor Research Labs Private Limited, Cyber City, Gurgaon 122002, India

Bashor et al. (1) very nicely demonstrate the importance of specific mutations in the switching of severe acquired respiratory syndrome coronavirus 2 (SARS-CoV-2) between in vivo and in vitro. They also identify 14 mutations that emerge when SARS-CoV-2 infects the animal species they study. However, their data analysis regarding low-frequency variants needs significant modification. Correct data analysis of low-frequency intrahost variants is also crucial for the calculation of transmission bottlenecks and associated disease mechanisms (2, 3).

The learned authors of ref. 1 claim that viral titer does not matter for how much variant richness is found. As evidence, they quote selected examples where low titer samples and high titer samples have similar numbers of variants (for instance, dogs vs. specific cats and hamster 1 vs. hamster 3). This claim is not tenable. In Fig. 1*A* (4), as high-depth samples are down sampled, the number of intrahost–single nucleotide variants (iSNVs) detected first increases and after reaching a peak, decreases. The samples quoted by Bashor et al. (1) could just be on different sides of the hump, or some samples could be on lower peaks and other samples on the sides of other curves, thus giving an illusion of invariance of the iSNV count with change in titer.

**Fig. 1.**
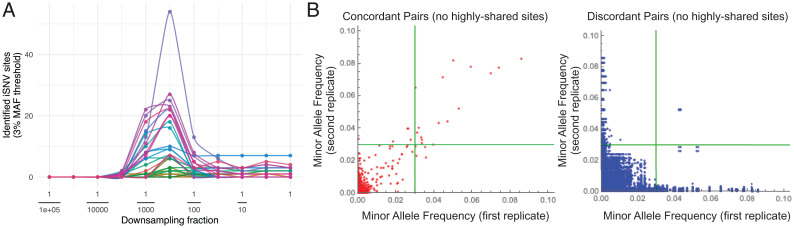
The number of iSNVs detected with different dilutions and frequencies. (*A*) High-depth samples from nasopharyngeal swabs of COVID-19 patients were down sampled, and the number of iSNVs identified above the 3% minor allele frequency (MAF) threshold is plotted for different dilutions. Different curves represent different samples. Bashor et al. (1) do not specify their frequency threshold, but even a different threshold is likely to give similar curves. (Figure 1A of ref. 4 has a plot of the number of iSNVs found in lower titer samples without dilution.) Reproduced with permission from ref. 4. (*B*) MAF is plotted for two replicates against each other for high-depth samples from nasopharyngeal swabs of COVID-19 patients. For concordant pairs, the data points are expected to be along the 45° line, while for discordant pairs, they are expected to be on one of the axes. Clearly, below an MAF of 3%, the data are nearly the same as noise. (Figure S8 of ref. 4 shows the necessity of a threshold of around 3% at high titers.) Also, that ref. 1 and ref. 4 use different primers should not matter for the broad trends. Reprinted with permission from ref. 4, which is licensed under CC BY 4.0.

Moreover, there needs to be a certain minimum frequency threshold of, say, 3% for identifying iSNVs even at very high viral titers. As shown in Fig. 1*B*, iSNVs below about 3% frequency are almost the same as noise. Therefore, claims of 564 unique variants above 0.1% frequency (and its comparison with cell culture) are not tenable—the default threshold of 3% was actually required even for high titer samples. Similarly, regarding the claim of 10 of 14 emergent variants being present between 0.1 and 3% frequency in the viral inoculum (thus suggesting in vivo selection of preexisting quasispecies), only some of these variants in the inoculum may be real.

Presence in both technical replicates, even if above the 3% threshold, is not always enough to claim genuineness of iSNVs (Fig. 2 has an explanation) (5). Thus, the claims of 88 unique variants (SNVs and structural variants) in 3 to 100% of the sequences and their observation 270 times need more careful analysis; low titer samples, like dogs and hamster 3, require much higher thresholds than 3% to prevent false iSNVs from getting included.

**Fig. 2.**
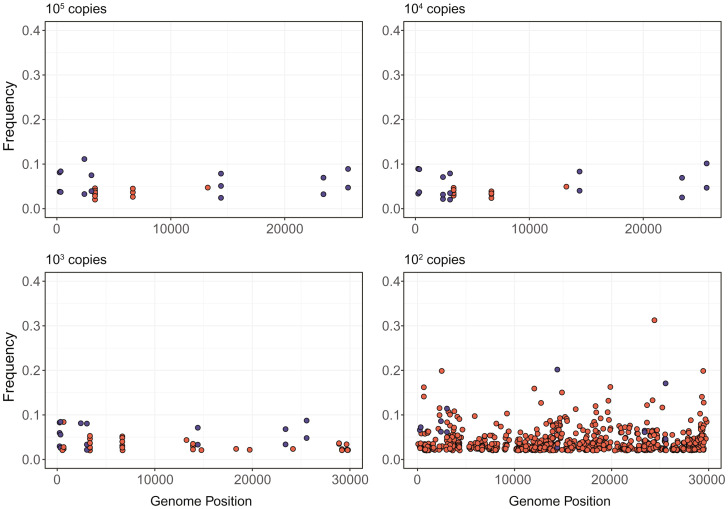
Frequencies of true iSNVs (blue) and false iSNVs (red) at different genome locations in a sequencing experiment with controlled mixtures of RNA sequences of SARS-CoV-2 genomes and RNA sequences of variant-containing SARS-CoV-2 genomes (ref. 5 has details of the experiment). Different subplots are for samples with different viral genome copies per microliter. At lower titers, false iSNVs are present at significantly higher frequency. (Extrapolating to Fig. 1*B*, for lower titers the noise region will expand significantly, requiring a threshold much higher than 3%.). Reprinted with permission from ref. 5, which is licensed under CC BY 4.0.

The sequences then not clustering by species may just be due to the large number of (random) false iSNVs included. This is the same for the uneven distribution of variants among different individuals of the same species. After excluding the variants in the viral inoculum, a large fraction of the variants were present in only one individual of a species; this fraction was much higher in dogs and lowest in cats, which is again consistent with the titers and thus, expected numbers of false iSNVs.

The differences in the levels of variant diversity from Lythgoe et al. (4) and Valesano et al. (5) are not due to species difference. The difference would likely be explained by the above checks and controls in the data analysis.

## References

[1] L. Bashor , SARS-CoV-2 evolution in animals suggests mechanisms for rapid variant selection. Proc. Natl. Acad. Sci. U.S.A. 118, 10.1073/pnas.2105253118 (2021). PMC8612357 34716263

[2] S. Gutiérrez, Y. Michalakis, S. Blanc, Virus population bottlenecks during within-host progression and host-to-host transmission. Curr. Opin. Virol. 2, 546–555 (2012). 22921636 10.1016/j.coviro.2012.08.001

[3] A. S. Lauring, Within-host viral diversity: A window into viral evolution. Annu. Rev. Virol. 7, 63–81 (2020). 32511081 10.1146/annurev-virology-010320-061642 PMC10150642

[4] K. A. Lythgoe ; Oxford Virus Sequencing Analysis Group (OVSG); COVID-19 Genomics UK (COG-UK) Consortium, SARS-CoV-2 within-host diversity and transmission. Science 372, eabg0821 (2021). 33688063 10.1126/science.abg0821 PMC8128293

[5] A. L. Valesano , Temporal dynamics of SARS-CoV-2 mutation accumulation within and across infected hosts. PLoS Pathog. 17, e1009499 (2021). 33826681 10.1371/journal.ppat.1009499 PMC8055005

